# Of Nature and Art in the Cure of Disease

**Published:** 1858-12

**Authors:** 


ART. VIII.— *Of Nature and Art in the Cure of Disease.* By Sir JOHN FORBES, M. D., D. C. L., (Oxon), F. R. S., Fellow of the Royal College of Physicians; Physician to the Queen's Household, etc., etc. From the second London Edition. New York: SAMUEL S. & WILLIAM WOOD, 389 Broadway. 1858.

The above is the title of a sprightly little work of two hundred and sixty duodecimo pages, issued lately by the enterprising house of the Woods of New York. "Nature and Art in the Cure of Disease," is a suggestive title. What is disease? what is its natural or unassisted termination? When we can answer these questions, we can say what nature has to do in the cure of disease; then the history of case of disease under the most approved medical treatment will tell us what Art can do in the same cause. We have been too much in the habit of regarding disease as an enemy with whom it was necessary for the doctor to immediately enter into deadly battle; assailing him generally with the lancet at first; then for fear of a recovery from this *coup*, following up the assault with calomel and jalap, blisters, seatons, etc., etc., in fearful succession, until finally the disease is vanquished, but alas, the patient whose delicate organism has been the field of this tremendous conflict, has been compelled to yield to the effects of the war between disease and the doctor; and though the latter is triumphant and the enemy is gone, he himself is no more. Then, indeed, can the bereaved friends say that "every thing has been done," and the probability is that an examination of the saddle bags of the valiant practitioner would indicate indeed that he has done all that was in his power.

This has too often been the history of the treatment of diseases, especially those of an acute character; and even now, some of our patients themselves are not satisfied if such vigorous measures be not employed. The great difficulty has been to know the *natural history* of disease, and now that we are tolerably well informed on this point, we know that many which have hitherto demanded the most active medication, will run a certain course and terminate of themselves in recovery. Pneumonia, which was formerly always treated by blood-letting, etc., is now proved to require little medication, and this is only one of many instances which modern researches have brought to light.

The author of the work before us has given us a familiar view of this subject, and his remarks are more particularly directed to the young practitioner. He reviews the general notions of diseases, their cause, nature, and mode of propagation. This part of the subject we pass over and come to the "course of diseases."

Here the author gives us an outline of two of the most common and important of our acute diseases, i. e., pneumonia and typhus fever. He follows, for example, the disease pneumonia, through all its phases. The exposure which may have been supposed to have induced it; the chills; the onset of the fever; quickened respiration and cough; rusty expectoration; and the evidence afforded by physical signs. Passing then to the pathological conditions of the affected part, he proceeds to give a brief description of them and to show how nature, after these structural changes have been effected, "sets her agents to work to undo her previous proceedings." The expectoration, instead of being diminished, is increased in quantity; this, indeed, being one of the means by which the morbid products are eliminated. Will our readers not acknowledge that this is about the history of most cases of simple pneumonia, confined, as it usually is, to a single lobe of one of the lungs? And is it probable that the course of treatment, unless it be of the most energetic kind, has much to do with this, the natural progress and termination of the disease? Cases which result unfavorably, or are of a longer duration, are variations from this healthy process, if we may so term it. Late observations have shown that venesection, which has always been the thought indispensable in acute pneumonia, has no effect in shortening the duration of the disease. The most enlightened treatment of pneumonia, then, in ordinary cases, unmodified by vice of constitution or endemic influences, is purely palliative; ease and comfort are secured to the patient by mild remedies; the disease is allowed to run its course, and the patient recovers. Here we have an instance where a knowledge of the natural history of a disease enables us to be mere spectators, palliating, it is true, this and that symptom, but not giving nature any decided aid. Here art merely looks at the operations of nature. But there are diseases where art has something more to do; when it is not enough not to interfere with the processes of nature, but it must assist her: nature is, perhaps, incapable of doing all the work, and a judicious interference on the part of the practitioner, enables her to shake off the disease at the proper time, and our patient recovers. In no diseases is art so competent to assist nature as in typhus and typhoid fevers. Here the concentrated nourishment and the stimulus keep the vital forces alive, and enable them to eliminate the morbific element, and after the passage of the disease, to rally and regenerate themselves. These facts are now becoming widely spread, and are so common that they actually are beginning to be known out of the profession.

The work of Sir John Forbes will do much good in presenting a review of this phase of medical science. It may serve to teach some that there are diseases which will cure themselves, and that the good results which are ascribed to a great variety of methods of treatment are merely due to this fact. When we have innumerable remedies for a certain disease, all with good evidences of success, we may often come to the conclusion, without further investigation, that we have here a self-limited disease. For example, how numerous are the specifics for inflammatory rheumatism: cases are are treated in the greatest variety of ways, and they all recover at about the same period. The author quotes, with reference to the disease, the pithy answer of Dr. Warren, when asked what would cure an acute rheumatism, "*Six weeks*," said Dr. Warren; and truly most rheumatic fevers run a course of six weeks and recover under almost any kind of treatment.

We commend the energy of the publishers in issuing works like the one before us, or Mind and Matter, which we reviewed in the last number. Such books do good. They are a relief from the dull systematic treatises, or the elaborate monograph, and lead the mind of the medical man out of the ordinary routine, to think for itself. Every one should read them, and if he does not find specific directions for the treatment or diagnosis of any one disease, he will get new ideas and suggestions which will make him take a more enlightened view of our science.

This work has been gotten up in handsome style by the Messrs. Wood, who will send it to any one in any part of the United States, free of postage, on the receipt of the price, which is $1.

